# A hierarchical analysis of associated factors to lifetime suicide attempts in alcohol use disorder

**DOI:** 10.47626/2237-6089-2024-0788

**Published:** 2025-12-05

**Authors:** Milton Moçambique, Daniela Benzano, Lisia von Diemen, Felix H. P. Kessler, Jaqueline B. Schuch, Flavio Pechansky

**Affiliations:** 1 Universidade Federal do Rio Grande do Sul Hospital de Clínicas de Porto Alegre Centro de Pesquisa em Álcool e Drogas Porto Alegre RS Brazil Centro de Pesquisa em Álcool e Drogas, Hospital de Clínicas de Porto Alegre, Universidade Federal do Rio Grande do Sul (UFRGS), Porto Alegre, RS, Brazil.; 2 Programa de Pós-Graduação em Psiquiatria e Ciências do Comportamento Porto Alegre RS Brazil Programa de Pós-Graduação em Psiquiatria e Ciências do Comportamento, UFRGS, Porto Alegre, RS, Brazil.

**Keywords:** Suicide, alcohol, childhood trauma, mental disorders, impulsivity

## Abstract

**Objective:**

Substance use is strongly associated with suicide attempts (SA) throughout life. We aimed to investigate the prevalence of SA and associated factors in life in patients with alcohol use disorder (AUD).

**Methods:**

The sample consisted of 424 men with AUD who underwent treatment at the Addiction Psychiatry Unit of the Hospital de Clínicas de Porto Alegre. The clinical evaluation included the Addiction Severity Index (ASI), the Childhood Trauma Questionnaire (CTQ), the Barratt Impulsiveness Scale (BIS), and the Structured Clinical Interview for the Diagnostic and Statistical Manual of Mental Disorders (SCID). Variables that showed p < 0.05 in the bivariate analyses were included in a hierarchical regression model with robust variance to investigate associations with SA in life via estimation of adjusted prevalence ratios (PR).

**Results:**

The lifetime prevalence of SA among alcohol users was 36.6%. Patients with childhood maltreatment, borderline personality disorder (BPD), and higher impulsivity scores had a higher lifetime prevalence of SA.

**Conclusion:**

Our findings indicate that male inpatient alcoholics present an important prevalence of SA. The study also corroborates the relationship between maltreatment, impulsivity, and BPD with SA among inpatients in treatment for AUD.

## Introduction

Suicide behavior is a severe global public health problem, primarily due to increased incidence rates, becoming one of the leading causes of death worldwide.^1^ This behavior is defined as any act by which an individual causes injury to him/herself, regardless of the degree of lethality,^2^ and comprises suicide ideation, suicide attempt (SA), and suicide.^3^ A previous SA is the most important risk factor for suicide to be considered in the general population.^4^

Several factors have already been associated with suicide risk. A systematic review conducted in the elderly population showed that the risk factors most associated with SA were depressive disorders, self-harm, and use of psychotropic drugs, followed by psychological and social factors, such as lack of social support and unemployment.^5^ A population-based study conducted in Japan on young people aged 13 to 18 showed that bullying and stress linked to family relationships were strongly associated with suicide tendencies.^6^ Moreover, a cohort study indicated that childhood trauma increased the lifetime risk of SA by two to five times^7^ Another recent study found that childhood trauma is highly associated with SA in several psychiatric disorders, such as bipolar disorder, depression, and schizophrenia.^8^ In cocaine users, childhood maltreatment was also related to suicide risk.^8,9^ In addition, in cocaine users of both sexes from Brazil, SA was also associated with depression and hallucinations, aggression, and severity of substance use. In a sample of Ethiopian substance users aged 15-24 years, suicide behavior was related to the female gender, poor social support, and the presence of anxiety.^10^

Studies indicate that substance use is strongly associated with SA throughout life. In this sense, the use of alcohol is also considered a significant risk factor for suicidal behavior.^3^ A meta-analysis found a significant relationship between alcohol use disorder (AUD) and suicidal ideation, attempted suicide, and completed suicide.^11^ The alcohol-suicide relationship should be understood through two distinct constructs: acute alcohol use and AUD (dependence).^12^ Heavy alcohol use is related to an increased risk of suicidal behavior and self-harm.^13-17^ Acute alcohol intoxication may increase the risk of suicidal behavior due to impaired critical judgment and loss of behavioral inhibition.^18-20^ Studies indicate that moderate alcohol consumption is associated with the use of more lethal methods for SA.^20,21^ Furthermore, alcohol is the most vital predisposing factor for impulsive and aggressive behavior.^22^

Suicidal ideation has also been associated with impulsivity in substance users.^23-25^ One study pointed out that individuals who had attempted suicide one or more times during their lifetime – in addition to greater impulsivity – had a more frequent history of alcohol abuse than individuals without previous SA.^26^ Anxious and impulsive personality traits^20,27^ were also linked to alcohol consumption and suicide.

Evidence also indicates a strong relationship between other psychiatric comorbidities in AUD patients and suicide. In hospitalized patients with AUD, depression was associated with an increased risk of suicide.^21^ A retrospective study that evaluated the risk factors associated with suicide in 50 male subjects with AUD showed that 76% had psychiatric comorbidities, and 72% had depression.^22^ This same study showed that most individuals also presented no social support, were unemployed, had health problems (liver cirrhosis, acute myocardial infarction, pulmonary emphysema, diabetes mellitus, and malignancy), and 38% lived alone. All these factors increase the risk of suicide.^22^

Based on the assumption of the suicide-stress diathesis model,^26^ we sought to identify, among individual suicide risk factors (childhood trauma; impulsivity; major depressive episode or major depressive disorder; hypomanic episode or bipolar disorder I and II; schizophrenia; borderline personality disorder [BPD]), which might be more associated with SA in patients with AUD. As far as we know, there are no studies evaluating SA and associated factors in alcohol users in Brazil, especially in the state of Rio Grande do Sul, which has been identified with the highest rates of suicidal behavior.^28-32^ This study could provide theoretical support for the mental health area in the development of public policies, suicide prevention actions, and the individualized management of suicidal behavior in health services. Due to the complexity of evaluating multiple risk factors simultaneously, we opted for a hierarchical model, to better structure the criteria and select the factors. Therefore, this study hypothesized that a history of childhood trauma, mental disorders, and impulsivity would be associated with a higher prevalence of suicide risk among inpatients in treatment for AUD.

## Methods

### Design and sample

This was a cross-sectional study, with retrospective data evaluation of a consecutive sample of men with AUD admitted to the Addiction Psychiatry Unit Addiction Psychiatry Unit of the Hospital de Clínicas de Porto Alegre. Data were collected by previously trained and supervised junior health researchers. Data collection occurred in the 1st week after the moment of hospitalization, in the period between 2012 and 2020.

The inclusion criteria were age between 18 and 65 years, diagnosis of AUD according to the Diagnostic and Statistical Manual of Mental Disorders, 4th edition (DSM-IV) or 5th edition (DSM-5),^33,34^ and absence of cognitive deficits that could impair the reliability of the responses through the Mini-Mental State Examination (MMSE).^35,36^ All participants voluntarily agreed to participate in the study and signed the informed consent form. Individuals who had difficulty understanding the questions in the research questionnaires and those who presented severe cognitive deficits when assessed by clinical impression were excluded from the sample. In total, 30 patients were excluded from the analyses because they had not completed the research protocol. The final sample consisted of 424 inpatients.

The SA was assessed based on self-report, considering the answers obtained through the questionnaire of sociodemographic data and the Addiction Severity Index-6 (ASI-6).^37,38^ The research protocol also included the following questionnaires: 1) Sociodemographic data questionnaire, with data on age, employment status, race/color, marital status, and family history of 1st-degree of drug dependency; 2) ASI-6, using the variables related to alcohol use; 3) Childhood Trauma Questionnaire (CTQ), where five components were analyzed (physical abuse, emotional abuse, sexual abuse, physical neglect, and emotional neglect)^39,40^; 4) Barratt Impulsiveness Scale (BIS-11), in the three components (motor, attentional impulsivity, and lack of planning)^41,42^; 5) Structured Clinical Interview for DSM Disorders (SCID)-I and II,^33,43^ to assess psychiatric and personality disorders.

### Statistical analysis

The lifetime prevalence of SA is presented with its respective 95% confidence interval (95%CI). Demographic data are compared between the groups: (a) patients with self-reported SA in their lifetime; and (b) patients who do not report SA in their lifetime. Quantitative variables are assessed for normality by the Kolmogorov-Smirnov test. Variables with normal distribution are described by the mean and standard deviation (SD) and compared between the groups by Student's *t* test for independent samples. Asymmetrical variables are described by the median and interquartile range (IQR) and compared by the Mann-Whitney *U* test. Categorical variables are described by frequencies and percentages and are associated with the chi-square test.

The prevalence of SA in individuals with AUD was assessed according to the severity of childhood trauma, impulsivity scores, and presence of psychiatric disorders. There are several statistical models to evaluate the prevalence ratio (PR), however considering the cross-sectional design of the study, and the number of potentially associated variables, previous studies have indicated the use of Poisson regression with robust variance as the most appropriate statistical approach.^44,45^ Therefore, a Poisson regression model with robust variance, hierarchical in blocks, and adjusted by age was performed to evaluate the PR of SA according to risk factors. Blocks were determined based on the biopsychosocial model for suicide.^46^ This model states that sociological, demographic, economic, and environmental factors can influence any or all of the distal developmental and proximal factors for suicide. Considering this scenario, a hierarchy was established for all risk factors that proved to be statistically significant in the bivariate analyses (p < 0.05): block 1 – childhood abuse; block 2 – mood disorders and personality disorders; and block 3 – impulsivity. All analyses were performed using IBM SPSS software (version 20), considering a 5% significance level.

### Ethical considerations

This is a secondary analysis of the project approved by the Research Ethics Committee of Hospital de Clínicas de Porto Alegre under protocol 2014-0249. The hypothesis tested is aligned with one of the project's specific objectives.

## Results

In a sample of male inpatients in treatment for AUD, the lifetime prevalence of SA was 36.6%. AUD patients with lifetime SA were younger than patients without SA. Occupation, marital status, education, skin color, and family history of substance use were similar between the groups (Table 1).

**Table 1:** Sociodemographic data and family history of substances in individuals with or without SA

Characteristic		No SA in life (n = 269)	With SA in life (n = 155)	p-value
Age		51.6 ± 9.3	49.1 ± 10.1	0.010
Occupation				
	Employed	80 (40.0)	40 (30.8)	
	Unemployed	59 (29.5)	52 (40.0)	0.107
	Retired	61 (30.5)	38 (29.2)	
Marital status				
	Single	186 (67.7)	116 (75.3)	0.258
Schooling				
	Elementary school complete	166 (63.1)	105 (68.2)	0.347
Skin color				
	White	170 (63.2)	90 (58.1)	0.346
Family history of substance use				
	Yes	148 (77.9)	98 (77.9)	0.815

95%CI = 95% confidence interval; SA = suicide attempt.

Variables by mean ± standard deviation (SD) or frequency (%). Sample size may show small differences due to losses for each variable. Significance level p ≤ 0.05.

Individuals with AUD with moderate to severe childhood trauma, including emotional and physical neglect, emotional and physical abuse, and sexual abuse, had a higher prevalence of SA compared to those with mild or no trauma (p < 0.05) (Table 2). Similarly, individuals with psychiatric disorders, such as depressive episodes, major depressive disorder, bipolar disorder type 1, and BPD, had a higher prevalence of SA than those without these disorders (p < 0.05) (Table 2). Higher scores of attentional, motor, and unplanned impulsivities were observed in individuals with SA compared to those without any reported lifetime SA (attentional 20.7 ± 3.6 versus 19.4 ± 3.5, p = 0.004; motor 25.1 ± 4.7 versus 22.3 ± 5.2, p < 0.001; and unplanned 28.3 ± 5.5 versus 25.9 ± 5.9, p = 0.001).

**Table 2:** Prevalence of SA in individuals with AUD according to the presence of trauma in childhood and psychiatric disorders

Variable/Category		Prevalence of SA in life	p-value
Emotional neglect			
	Mild	76 (31.3)	0.029
	Moderate to severe	21 (50.0)	
Physical neglect			
	Mild	47 (27.5)	0.006
	Moderate to severe	50 (43.9)	
Emotional abuse			
	Mild	55 (27.2)	< 0.001
	Moderate to severe	41 (43.9)	
Physical abuse			
	Mild	51 (26.8)	< 0.001
	Moderate to severe	46 (48.4)	
Sexual abuse			
	Mild	73 (29.6)	< 0.001
	Moderate to severe	23 (63.9)	
Schizophrenia			
	Absence	94 (34.2)	0.571
	Presence	2 (66.7)	
Major depressive episode			
	Absence	28 (26.4)	0.033
	Presence	69 (39.7)	
Major depressive disorder			
	Absence	67 (30.2)	0.003
	Presence	29 (52.7)	
Bipolar affective disorder type 1			
	Absence	91 (33.5)	0.014
	Presence	6 (85.7)	
Bipolar affective disorder type 2			
	Absence	92 (34.3)	0.663
	Presence	5 (45.5)	
Borderline personality disorder			
	Absence	48 (33.3)	0.002
	Presence	17 (68.0)	

95%CI = 95% confidence interval; AUD = alcohol use disorder; SA = suicide attempt.

Variables by mean ± standard deviation (SD) or frequency (%). n values may present differences due to the losses of each variable. Significance level p ≤ 0.05.

The Poisson regression model with robust variance showed that the presence of mood disorders was not associated with increased lifetime SA in individuals with AUD (PR 1.49; 95%CI 0.89-2.50; p = 0.127). On the other hand, the presence of moderate to severe childhood trauma (PR 1.75; 95%CI 1.21-2.54; p = 0.003), BPD (PR 1.82; 95%CI 1.17-2.82; p = 0.008), and higher impulsivity scores (PR 1.03 95%CI 1.01-1.05; p = 0.011) were associated with an increased prevalence of lifetime SA (Table 3).

**Table 3:** Hierarchical Poisson regression with robust variance for SA and PR

	PR	95%CI	p-value
Moderate to severe maltreatment	1.75	1.21-2.54	0.003
Mood disorders	1.49	0.89-2.50	0.127
Borderline personality disorder	1.82	1.17-2.82	0.008
Impulsivity	1.03	1.01-1.05	0.011

95% CI = 95% confidence interval; PR = prevalence ratio; SA = suicide attempt.

Significance level p ≤ 0.05. Age-adjusted analyses.

## Discussion

Our results demonstrate a very high prevalence of SA among male inpatients with AUD. The presence of moderate to severe maltreatment, BPD, and higher impulsivity scores were associated with an increased in the lifetime prevalence of SA in this sample of users.

Suicidal behaviors are known to be a typical symptom of BPD.^33,47^ In clinical populations, the suicide rate of patients with this disorder is estimated to be between 8 and 10%.^48,49^ Studies indicate that patients with BPD and substance abuse problems have a poorer prognosis and are at a greater risk of suicide or death by injury or accident.^50,51^ In the present study, the lifetime prevalence of SA in alcohol users with BPD was 68%, with a PR of 1.82. Our findings are in line with previous studies, showing that BPD is also a risk factor for suicide in AUD, deserving greater attention in preventive approaches, as well as in public health policies. BPD presents alterations in a few cognitive domains such as executive functions, attention, concentration, working memory, decision-making, and impulse control. In BPD patients with a history of SA, deficits in executive functions are even more pronounced, generating high risks for both suicide and an increase in the number of attempts. The relevance of assessing cognitive aspects, impulsivity, and suicide risk in BPD in individuals with AUD lies in the possibility of generating data to improve forms of intervention in this disorder, especially to prevent suicidal behavior and the serious risk of early death.

Our findings showed that the presence of moderate to severe childhood abuse was related to an increased prevalence of suicide in our sample of AUD patients, in agreement with previous data.^7,27^ Studies have shown an association between childhood trauma and drug addiction with the risk of SA later in life.^46,52,53^ A study of male cocaine users found that childhood trauma was significantly associated with suicidal behavior.^9^ Childhood maltreatment should be considered an important risk factor for attempted suicide in individuals with AUD. The consequences of childhood maltreatment should encourage us to identify those at risk early and to develop effective interventions to protect children from violence.

In conceptual terms, impulsivity is a predisposition to act immediately, an unplanned reaction to internal (stress) or external stimuli, without considering the consequences for others or oneself.^45^ Our results are similar to those described in the literature,^23-26^ in which impulsivity was associated with a higher prevalence of SA. It is complex to dissociate impulsivity as an isolated risk factor in this scenario. However, it is important to highlight that the presence of a history of trauma in childhood is associated with a great predisposition to develop BPD and impulsive behavior, adding to the risk factors that contribute to suicidal behavior.

Several studies^22,26,54^ point to mood disorders as the main risk factor for suicide. Still, our findings differ, and mood disorders were not associated with an increased prevalence of suicidal behavior in AUD patients. However, it is important to emphasize that there is no scientific study that can infallibly predict the occurrence of suicide in patients with depression. Given that no single risk factor explains suicidal behavior, our findings cannot be interpreted as contradictory. It is understood that, at a given time, a risk factor presents in the individual's life^8,23-25^ might be a determining factor for suicidal behavior.

This study has some limitations. Data are based on retrospective self-report of SA, and individuals may have under or misreported data as a function of memory biases, especially as this was a sample of subjects with AUD undergoing detoxification. Our sample comprised only men, making it impossible to generalize the results for women. Furthermore, the study design does not allow for causal inferences between predictors and suicide but explores the relationship between variables. These limitations are due to the use of secondary databases, since the factors associated with SA were limited to the variables available in the data collection instruments. Longitudinal studies are preferable to examine the risk factors for SA better, considering different contexts and the specificity of each risk factor.

## Conclusion

In summary, our findings indicate that individuals with AUD present an important prevalence of SA. The study also corroborates the relationship between moderate to severe childhood maltreatment, impulsivity, and BPD with SA. In this sense, it is necessary to develop specific preventive and therapeutic strategies and effective public policies, considering all these factors in this vulnerable population.

## Data Availability

The data that support this study are available from the authors upon request.
